# The benefits of co-location in primary care practices: the perspectives of general practitioners and patients in 34 countries

**DOI:** 10.1186/s12913-018-2913-4

**Published:** 2018-02-21

**Authors:** M. Bonciani, W. Schäfer, S. Barsanti, S. Heinemann, P. P. Groenewegen

**Affiliations:** 1 Laboratorio Management e Sanità, Institute of Management, Scuola Superiore Sant’Anna, Pisa, Italy; 2 Netherlands Institute for Health Services Research-NIVEL, Utrecht, The Netherlands; 3 Department of General Practice, University Medical Center Göttingen, Göttingen, Germany; 4 Department of Nursing and Health Sciences, University of Applied Sciences Fulda, Fulda, Germany; 5 Department of Sociology, Department of Human Geography, Utrecht University, Utrecht, The Netherlands

## Abstract

**Background:**

There is no clear evidence as to whether the co-location of primary care professionals in the same facility positively influences their way of working and the quality of healthcare as perceived by patients. The aim of this study was to identify the relationships between general practitioner (GP) co-location with other GPs and/or other professionals and the GP outcomes and patients’ experiences.

**Methods:**

We wanted to test whether GP co-location is related to a broader range of services provided, the use of clinical governance tools and inter-professional collaboration, and whether the patients of co-located GPs perceive a better quality of care in terms of accessibility, comprehensiveness and continuity of care with their GPs. The source of data was the QUALICOPC study (Quality and Costs of Primary Care in Europe), which involved surveys of GPs and their patients in 34 countries, mostly in Europe. In order to study the relationships between GP co-location and both GPs’ outcomes and patients’ experience, multilevel linear regression analysis was carried out.

**Results:**

The GP questionnaire was filled in by 7183 GPs and the patient experience questionnaire by 61,931 patients. Being co-located with at least one other professional is the most common situation of the GPs involved in the study. Compared with single-handed GP practices, GP co-location are positively associated with the GP outcomes. Considering the patients’ perspective, comprehensiveness of care has the strongest negative relationship of GP co-location of all the dimensions of patient experiences analysed.

**Conclusions:**

The paper highlights that GP mono- and multi-disciplinary co-location is related to positive outcomes at a GP level, such as a broader provision of technical procedures, increased collaboration among different providers and wider coordination with secondary care. However, GP co-location, particularly in a multidisciplinary setting, is related to less positive patient experiences, especially in countries with health systems characterised by a weak primary care structure.

## Background

The co-location of professionals in primary care settings involves a structural change in healthcare provision in many countries where traditionally general practitioners (GPs) work in single-handed practices, and in these contexts co-location is adopted to improve primary care, in pilot areas or more widely [1, 5, 7]. The co-location of GPs with other professionals may positively change their way of working and may also improve the quality of healthcare as perceived by patients. In this article, we analyse how GP co-location relates to the experiences of GPs and patients.

Co-location involves the logistic integration of professionals working together in the same facility. In our study, it has been defined through two dimensions: i) mono-professional co-location of GPs (usually with secretarial support); ii) multi-disciplinary co-location. Most of the current literature focuses on multi-disciplinary co-location.

In the West, there is considerable variation in the composition of primary care teams, with large differences in the mix of professions working with GPs within the same practice [22]. This may be related to the level of attention national policies give to co-locating GPs with other professionals as a strategy to improve integration in primary care. In fact, the co-location of GPs and other professionals in the same practice, as a single point of healthcare access, is often proposed as a change in the organisation of primary care delivery that may facilitate access to services. This co-location may also minimize fragmentation among the various providers involved in the patient care pathway, by reducing duplication and ensuring more responsiveness to users [9, 24, 40]. This is particularly important for patients with chronic conditions or multi-morbidity [6, 55], who require a comprehensive approach and the involvement of different professionals in the provision of integrated care [20, 54]. The co-location of services and professionals in primary care can facilitate integration in the delivery of healthcare, prevention and rehabilitation services [48].

Professionals who are co-located in the same facility have more opportunities to meet and share information. Such increased interaction in co-located settings can enhance a mutual influence in decision-making and clinical practice [13]. This mutual influence and the need for coordination following the increased interactions are potentially important in terms of clinical governance. For instance, guidelines are more readily accepted as a result of local consensus discussions and contact with colleagues [23].

However, it has also been argued that professionals may consider that a mutual influence related to teamwork could negatively impact on their autonomy [8]. Consequently, GPs may oppose organisational changes that potentially promote teamwork, such as co-location in the same facility.

Co-location provides opportunities for collaboration but does not necessarily lead to it. There is mixed evidence on the role of co-location as a key driver for integration among professionals [39]. Many studies have shown that co-location facilitates multi-professional teamwork [11, 57] and the possibility to share information on patients and jointly define their care pathways [4, 12]. However, co-location by itself has been shown to not necessarily lead to collaboration among professionals when they continue to work as separate providers [27, 36].

How the co-location of primary care professionals relates to patients’ experiences is not yet clear. The underlying assumption is that a greater degree of organisational integration resulting from co-location benefits patients. Some studies have reported that the key aspect characterising co-location is joint working, which promotes better results for patients and an improvement in service quality, in terms of improved access to health services for patients and the increased satisfaction of patients [19]. However, other studies have highlighted that patients prefer single-handed or small practices [3], and that smaller practices are perceived as being more accessible [10].

Considering the controversial evidence, this study aims to analyse the relationship between GP co-location with other GPs and/or other professionals and the GPs’ outcomes and patients’ experiences. We expect that when GPs are co-located with other GPs and/or other professionals, they provide a broader range of services, use more clinical governance tools and collaborate more with other primary and secondary care professionals (GPs’ outcomes). We also hypothesise that the patients of co-located GPs with other GPs and other professionals perceive a better quality of care in terms of accessibility, comprehensiveness of care and continuity of care with their GPs (patients’ experiences). We expect that patients of GPs who are co-located with other GPs and/or other professionals, perceive a better quality of care by receiving more accessible care, with a more comprehensive approach which enables them to have answers to a broader range of common health problems and more continuity of information between primary and secondary care professionals, compared to patients of GPs in single-handed practices.

**Fig. 1:**
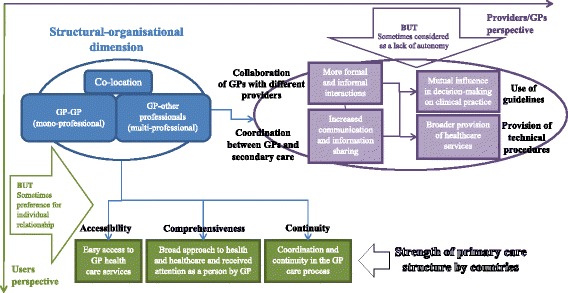
Relationships between GP co-location and GPs’ and patients’ experiences: conceptual framework of the study

Figure 1 summarizes the conceptual framework of this study. It shows how the structural and organisational dimension of healthcare, represented by GP co-location, can be related to different potential outcomes from the perspective of GPs and experiences from the perspective of patients. The two perspectives can sometimes be conflicted [38], however we hypothesise that both providers (GPs) and users (patients) converge in experiencing advantages from the GP-colocation with other GPs and/or other professionals.

Our study will therefore attempt to answer the following questions:

- What relationships are there between GP co-location and the range of services provided, the use of clinical governance tools and inter-professional collaboration?
- What are the relationships between GP co-location and the accessibility, comprehensiveness and continuity of care as perceived by their patients?
- Do the mono- and multi-professional co-location of GPs and the combination of these two dimensions relate differently to GP outcomes and patient experiences?

Mono-professional GP co-location is particularly interesting because, to the best of our knowledge, its role in terms of GP outcomes and patient experience has not yet been analysed. Regarding multi-professional GP co-location, we will also investigate the relationships between GP outcomes and patient experience and GPs co-located with nurses, other health professionals and social workers.

Finally, we consider the role of the strength of primary care at the national level [30, 31] in conditioning the relationships between GP co-location and patient experience, in fact a stronger primary care structure is associated with more accessible, continuous and comprehensive care [50].

Before presenting the methodological details, the following section overviews how GP co-location has functioned in the 34 countries involved in this study.

### Co-location in primary care: Some experiences in western countries

The term co-location is used for the organisational strategy of grouping different professionals in the same facility, which in primary care particularly involves GPs, nurses, other health professionals (such as midwives, physiotherapists, dentists, pharmacists or even specialists of secondary care) and social workers.

Although Groenewegen et al. [22] do not refer explicitly to the concept of co-location, they observed a high variability in the number of professionals working with GPs within the same practice. Of the 34 countries we analysed, almost half (16) are characterised by GPs working with at least three professionals. The largest number of co-located professions with GPs are in Lithuania (8 other professions), Finland (7), Cyprus and Spain (6) and Iceland, Sweden and England (with 5 other professions).

All these countries, where a considerable level of GP co-location is quite widespread, are very different in terms of health system characteristics and primary care development (see Appendix 1). The co-location strategy does not seem to be related particularly to specific health system characteristics. Indeed, although the majority are countries with a tax-funded national health system, there are also transitional countries (Lithuania, Poland, Slovenia), where GPs are co-located in large polyclinics typical of their previous healthcare system, and one country with a social health insurance system (i.e. the Netherlands, with the longest experience of primary care centres with GPs, nurses, social workers, physiotherapists).

Although the majority of countries have strong primary care with GPs playing a gatekeeping role, the two with the largest GP practice (Cyprus with urban and rural health centres, and Iceland with primary care centres located throughout the country) have the weakest primary care system, according to the Primary Health Care Activity Monitor Europe study [30–33]. However, a significant association was observed between stronger primary care systems, in particular with a better developed primary care workforce and more comprehensive primary care processes, and larger primary care practices with more primary care professions [22].

The experiences of co-location are country dependent and it is difficult to summarize any common policies regulating their implementation. In Lithuania, GPs are co-located in primary care centres and polyclinics which often provide both primary and secondary outpatient services. Finnish primary health centres offer a broad range of primary health care services at the municipality level. In Spain, GPs work together with nurses, social workers, paediatricians, midwives, specialists, dentists, physiotherapists, with a formalised primary care multi-professional team. GP-led health centres in England have a more limited inter-professional collaboration without the presence of social workers. In Sweden primary care is provided in multidisciplinary teams, with at least one GP and one nurse, but often with social workers, psychologists and physiotherapists working at the primary health care centres. Medical homes in Australia and New Zealand promote the person-centred medical home (PCMH) model, with patients having an ongoing relationship with a particular doctor (who leads a multidisciplinary practice team) and primary care that is comprehensive, coordinated and accessible, with a focus on safety and quality.

The key common aspect of all these situations is that GPs and other professionals work together in the same facility, which constitutes the variable of interest to be explored in relation to the GPs’ working methods and patient experiences.

## Methods

We used data derived from the QUALICOPC study (Quality and Costs of Primary Care in Europe). In this study, co-funded by the European Commission, surveys were conducted among GPs and patients in 31 European countries (EU 27 except for France, along with FYR Macedonia, Iceland, Norway, Switzerland and Turkey) and three non-European countries (Australia, Canada, New Zealand). The QUALICOPC study used nationally representative samples in the majority of countries. GPs and patients were surveyed with standardized questionnaires. One GP per practice was sampled. Target response was 220 GPs (75 in the four smallest countries). In every practice, nine patients filled in a Patient Experience questionnaire on the consultation that had just occurred. Ethical approval was acquired in accordance with the legal requirements in each country. Depending on the national requirements, written or oral informed consent was requested.

GPs were invited via letter, email or telephone and gave their consent to participate in the study. Patients were invited by the fieldworker or practice staff to complete a questionnaire. All participants were informed about the study and participation was voluntary [44]. A unique practice identification number anonymously linked GP responses to the responses of their patients, allowing for multi-level analyses of the data.

Data collection took place between October 2011 and December 2013. The GP questionnaire was filled in by 7183 GPs and the Patient Experience questionnaire by 61,931 patients (database version 4.1, June 2014). The age and gender of the participating GPs were compared to national statistics and the representativeness of these two variables was generally good [21]. Details regarding the study protocol and questionnaire development have been published elsewhere [21, 51, 52].

### Independent variables concerning GP co-location

The main explanatory factor included in this study is GP co-location which is defined as a GP located in the same primary care facility with other GPs and/or other professionals. GP co-location is measured by two dichotomised variables:

- GP alone or with other GPs, considered as mono-professional GP co-location;
- GP(s) with at least one other health or social care professional, considered as multi-professional GP co-location.

The two variables were created on the basis of the answers to the GP questionnaire, which contained questions asking whether or not a GP works in the same practice/centre with other GPs, specialists and twelve types of other professionals.

We defined multi-professional GP co-location as GP(s) co-located with a nurse or another health professional (specialist, midwife, physiotherapist, dentist or pharmacist) or social worker. For the nursing role, we considered practice nurses, community nurses and nurse practitioners. If nurses worked with GP(s) without a secretary/receptionist and if they did not provide vaccinations, health information, do check-ups or minor clinical activities, they were not considered as playing a real nursing role, but as a substitute for a secretary/receptionist. The health professionals co-located with a GP are those providing specialised care, maternal care, rehabilitation, oral health care or pharmaceutical services and contribute to comprehensive health care delivery [25, 29, 53]. Finally, multi-professional GP co-location also takes into account the presence of a social worker, who facilitates the integration of the social and health domains in primary care [16, 26, 56].

We investigated the association of the two different dimensions of GP co-location with GPs’ and patients’ experiences, both as a separate (additive) and joint effect (interaction). We also analysed the association between GPs’ and patients’ experiences and co-location of GP with specific professionals, such as nurses, other health professionals (specialists, midwives, physiotherapists, dentists or pharmacists) and social workers.

### Dependent variable: GPs’ outcomes and patients’ experiences

We studied the relationships between GP co-location and both GPs’ outcomes and patients’ experience.

At the GP level, we explored four dependent variables that we hypothesised would be associated with GP co-location. They concern GP clinical practice, in terms of health care provision, clinical behaviour and inter-professional working. The following GP variables were included:

- provision of technical procedures (sum score of 10 procedures),
- use of guidelines (sum score in four areas: chronic heart failure, asthma, COPD, diabetes),
- collaboration with other providers (3 categories of meeting frequency of GPs with 10 types of primary care professionals);
- coordination between GPs and secondary care (3 categories of meeting frequency of GPs asking for advice from 10 different types of specialists).

All previous outcomes were measured through scales constructed through latent class multilevel analyses with items nested in GPs and in countries [52].

The other group of independent variables concerns the experiences of patients. We used scales measuring accessibility (5 questions on the access to GP practices concerning organisational aspects, such as for instance opening hours), comprehensiveness (2 questions on whether GPs represent the first contact for common health problems) and continuity of care with their GPs (3 questions on whether GPs have known the patient on a long-term basis). These scales were constructed through latent class multilevel analyses with items nested in patients, GPs and countries and these had been used in other studies to measure patient perceptions of GP quality [50].

### Statistical analysis

To analyse the relationship between GP co-location and GP outcomes and patient experiences, a multilevel linear regression analysis was used, since all dependent variables can be treated as continuous variables considering the process used to build them. This study included 60,762 patients of GPs who had completed a questionnaire (7163 GPs).

When analysing outcome variables concerning GPs, the country represented the highest level and GP the lowest. A third level (patient) was included in the models analysing the patients’ experiences.

In order to answer the three research questions, we performed four models for each dependent variable, both at the GP and patient levels. In the first two models, we explored the main effects of the two components of GP co-location separately (mono and multi-professional). The results of these analyses are in the Appendix 3.

The third model focused on the interaction of the two variables concerning GP co-location. In this model, we found differences in the relationship with outcomes among four possible settings: 1) only one GP without other professionals (single-handed GP practice), 2) GP with only other GPs (i.e. no other types of professionals), 3) only one GP with other professionals and, 4) two or more GPs with other professionals (Tables 4 and 5). Finally, in the fourth model we considered the three sub-variables of multi-professional GP co-location separately, in order to highlight the specific relationships between co-location with nurses, with other health professionals and with social workers and GP outcomes and patient experiences (results in the Appendix 3).

The interpretation of the results of the four models applied to the GP outcomes enabled us to answer the first research question, while the second research question was answered by the four models applied to the patient experiences. The analysis of differences in the relationships between GP co-location and GP outcomes and patient experience emerging from the comparison of the results among the four models enabled us to answer the third research question (Table 1).

**Table 1:** Framework of the study methods

Methods used to answer the research questions		Dependent variables	
Multilevel regression models	Independent variables	GP outcomes (4 variables)	Patient experience (3 variables)
*Model 1*: Mono-professional co-location	*Variable 1*: GP with other GPs	*First research question*: What relationships are there between GP co-location and the range of services provided, the use of clinical governance tools and inter-professional collaboration?	*Second research question*: What are the relationships between GP co-location and the accessibility, comprehensiveness and continuity of care as perceived by their patients?
*Model 2*: Multi-professional co-location	*Variable 2*: GP with other professionals		
*Model 3*: Interaction between mono- and multi-professional co-location	*Variable 1 x Variable 2*: - Single-handed GP practice - Two or more GPs without other professionals - One GP with other professionals - Two or more GPs with other professionals		
*Model 4*: Details of GP co-location with multi-professionals	*Variable 2 detailed*: - GP with nurse - GP with other health professionals - GP with social worker		
Comparison of the results between the four models in order to identify differences in the relationships between independent variables and dependent variables		*Third research questions*: Do the mono- and multi-professional co-location of GPs and the combination of these two dimensions relate differently to GP outcomes and patient experiences?	

All models were adjusted for GP covariates, such as sex, age and location of practice in urban/rural areas and involvement in disease management. Evening opening times of the GP practice and its location were considered as GP covariates in the adjusted model that analysed patients’ experiences.

All the models concerning patients’ experiences were also adjusted for patients’ socio-demographic and health characteristics (sex, age, education, ethnicity, household income, self-reported health status and chronic conditions), the main reason for the visit to GPs (administrative vs health reason) and for having their ‘own’ doctor.

We reported the variances in the empty model, in the model with only the covariates, and in the model with the covariates plus the variables of interest (the combination of mono and multi-professional co-location).

As a sensitivity analysis, we tested whether the differences concerning the coordination with secondary care were still present when removing GPs who were co-located with specialists. This kind of sensitivity analysis verifies the specific role of specialists in the relationship between GPs co-located with other professionals and integration with secondary care.

We used the GP outcome variables (such as the provision of technical procedures, use of guidelines, collaboration with other providers and coordination between GPs and secondary care) and transformed them into categorised variables to perform stratified analyses in order to verify whether they had an interaction effect with co-location on patient outcomes.

Finally, to obtain more information to answer the second research question, we explored the possible differences in the relationships between GP co-location and patient experience at the country level, by performing a stratified analysis of the models concerning patient outcomes based on the level of strength of the primary care structure in each country [30–32]. The strength of primary care systems at a national level was categorized in tertiles.

All analyses were performed using Stata 14.

## Results

The median participation rate of GPs was 30% [21] and the average response rate of patients was 74% [50]. Table 2 reports the GP co-location in all 34 countries. The tables show the distribution of each separate independent variable: GP co-location with other GPs and GP co-location with other professionals, with the details of GP co-location with nurses, other health professionals, and social workers. The table highlights a large variability in the distribution of independent variables.

**Table 2:** Distribution of the independent variables by country

Countries	Type of GP co-location				
	GP with other GPs^a^	GP with other professionals			
		At least one^b^	Nurse	Health professional^c^	Social worker
	Practice (%) *N* = 6880	Practice (%) *N* = 7163	Practice (%) *N* = 7051	Practice (%) *N* = 7163	Practice (%) *N* = 7047
Austria	8.8	34.8	26.3	15.8	2.9
Belgium	46.9	13.0	8.0	8.0	6.7
Bulgaria	22.5	79.4	74.9	20.2	2.3
Cyprus	85.9	91.6	91.3	88.7	18.8
Czech Republic	11.0	95.9	96.3	5.5	0.0
Denmark	71.6	80.2	81.0	5.7	0.5
Estonia	31.0	100.0	99.2	20.2	1.6
Finland	65.1	99.3	99.7	87.3	45.4
Germany	38.2	27.3	22.7	6.3	0.0
Greece	45.5	72.7	69.1	47.7	19.1
Hungary	11.3	96.0	98.6	9.5	4.2
Iceland	97.5	100.0	97.5	98.8	20.0
Ireland	72.5	96.4	95.8	36.9	8.4
Italy	51.6	22.5	19.4	8.7	2.0
Latvia	9.8	99.5	99.5	9.6	3.7
Lithuania	53.4	100.0	99.6	93.8	48.0
Luxembourg	49.4	15.4	6.7	10.3	5.3
Malta	54.4	75.7	58.6	72.9	6.9
Netherlands	69.8	97.0	96.6	42.6	18.7
Norway	99.0	54.0	40.4	26.8	1.5
Poland	65.9	99.5	98.2	75.5	5.5
Portugal	. ^d^	100.0	100.0	19.4	42.6
Romania	53.6	94.1	93.6	20.0	3.2
Slovakia	3.2	91.4	91.3	5.0	1.4
Slovenia	17.5	98.5	98.1	63.6	4.4
Spain	94.6	100.0	99.3	83.1	77.5
Sweden	99.0	100.0	100.0	55.7	69.1
Switzerland	48.2	21.1	6.1	15.1	1.0
Turkey	88.0	99.0	94.7	76.6	0.3
UK (England)	92.9	100.0	100.0	78.1	3.0
Australia	88.2	88.8	84.6	44.1	4.7
Canada	79.7	69.7	60.2	37.9	21.8
New Zealand	87.4	98.2	98.2	47.0	6.0
FYR Macedonia	47.5	97.2	94.9	22.4	1.5
**Average**	**56.5**	**78.3**	**75.5**	**38.9**	**15.5**
Weak PC countries	36.2	78.6	75.2	32.4	6.0
Medium PC countries	53.0	71.0	65.5	39.1	11.9
Strong PC countries	72.3	84.9	82.3	42.9	23.2

^a^Percentage of the sampled GPs co-located with at least another GP, irrespective of whether other professionals are present or not

^b^Percentage of the sampled GPs co-located with at least another professional (nurse, health professional - specialist, midwife, physiotherapist, dentist, pharmacist -, social worker), irrespective of whether other GPs are present or not

^c^Health professionals include specialists, midwives, physiotherapists, dentists, pharmacists

^d^All values of this question are recoded into missing due to a translation error in the GP questionnaire for Portugal

Around half of the GPs work in the same practice/centre with other GPs. There are only nine countries where more than 85% of GPs are co-located with other GPs, including Iceland, Norway and Sweden with almost all GPs working in a co-located setting. On the other hand, in Austria, Latvia and particularly Slovakia less than one GP in ten is co-located with other GPs.

Being co-located with at least one other professional is the most common situation of the GPs involved in the study. Only in Belgium, Italy, Luxemburg and Switzerland is this type of GP co-location found in less than 25% of all practices. In some countries all GPs are co-located with other professionals (Estonia, Iceland, Lithuania, Portugal, Spain, Sweden, England), and many others exceed 90%.

Within multi-professional GP co-location, being in the same practice/centre with nurses is the most common, while only around one in three GPs are co-located with specialists, midwives, physiotherapists, dentists or pharmacists. Cyprus, Finland, Lithuania, Malta, Poland, Spain and England have the highest percentage of GPs co-located with other health professionals (around 75–85%), while Belgium, Czech Republic, Denmark, Germany, Hungary, Italy, Latvia and Slovakia have the lowest proportions (below 10%). Co-location of GPs with a social worker is even rarer (only 15%), however there are countries where GPs never work in the same practice/centre with social workers (Czech Republic, Denmark, Germany, Turkey). Spain and Sweden are outsiders because their GPs are very often co-located with a social worker, and in Lithuania, Finland and Portugal this is also quite common.

Countries with weak primary care [30, 31] have fewer GPs co-located with other GPs, other health professionals and social workers, while they have a higher percentage of GPs co-located with nurses compared to countries with a medium primary care structure. Countries with strong primary care most often show GP co-location (Table 2).

Considering the interaction of the two independent variables (Table 3), mono-professional GP co-location and single-handed GP practice are on the whole less common (respectively 9.6% and 12.9%) than multi-professional GP co-location with only one GP (30.7%) and with more GPs (46.9%).

**Table 3:** Interaction between the two independent variables

Types of GP co-location		GP with other professionals (multi-professional co-location)		Total
		No	Yes	
GP with other GPs (mono-professional colocation)	No	*1) Single-handed GP practice*	*3) One GP with other professionals*	*GP alone*
		886	2109	2995
		12.9%	30.7%	43.5% (cell)
		29.6%	70.4%	100.0% (row)
		57.2%	39.6%	43.5% (col)
	Yes	*2) More GPs without other professionals*	*4) More GPs with other professionals*	*GP with other GPs*
		662	3223	3885
		9.6%	46.9%	56.5% (cell)
		17.0%	83.0%	100.0% (row)
		42.8%	60.4%	56.5% (col)
Total		*GP without other professionals*	*GP with at least one professional*	*Total*
		1589	5291	6880
		22.5% (cell)	77.5% (cell)	100.0%
		22.5% (row)	77.5% (row)	100.0%
		100.0% (col)	100.0% (col)	100.0%

Detailed figures of GP outcome variables are provided in the Appendix 2, while the other patient outcome variables have been reported in other studies [50].

### GP outcomes

The multilevel regression analysis (Table 4) revealed that the majority of variation in GP outcomes is at the GP level, except for the provision of technical procedures which is mainly related to the country level (intraclass correlation, ICC, 69.2%). Coordination with secondary care and use of guidelines have the lowest ICC at the country level (respectively 13.4% and 20.6%), however, these are still relatively high ICCs.

**Table 4:** Relationships between GP co-location and GP outcomes

Multilevel models (*p < 0.05; **p < 0.01; ***p < 0.001)	Provision of technical procedures^a^		Use of guidelines^a^		Collaboration with different providers^a^		Coordination with secondary care^a^	
*Fixed part*	*Coeff.*	*S.E.*	*Coeff.*	*S.E.*	*Coeff.*	*S.E.*	*Coeff.*	*S.E.*
Single-handed GP practice (ref)								
- More GPs without other professionals	0.153***	0.023	0.021	0.013	0.033*	0.013	0.090***	0.024
- One GP with other professionals	0.146***	0.022	−0.003	0.012	0.106***	0.013	0.108***	0.022
- More GPs with other professionals	0.200***	0.021	−0.001	0.012	0.143***	0.012	0.080***	0.022
*Random part / Variance in full model*	*Var.*	*S.E.*	*Var.*	*S.E.*	*Var.*	*S.E.*	*Var.*	*S.E.*
Country level	0.415	0.100	0.012	0.003	0.023	0.006	0.028	0.007
GP level	0.182	0.003	0.053	0.001	0.057	0.001	0.184	0.003
Variance in empty model	Var.	S.E.	Var.	S.E.	Var.	S.E.	Var.	S.E.
Country level	0.452	0.110	0.014	0.004	0.025	0.006	0.029	0.007
GP level	0.201	0.003	0.054	0.001	0.060	0.001	0.186	0.003
*Variance in model only with covariates*	*Var.*	*S.E.*	*Var.*	*S.E.*	*Var.*	*S.E.*	*Var.*	*S.E.*
Country level	0.409	0.099	0.012	0.003	0.022	0.005	0.029	0.007
GP level	0.185	0.003	0.053	0.001	0.057	0.001	0.182	0.003

^a^Covariates GP level: sex, age, urbanisation, involvement in disease management

All types of GP co-location are positively associated with the provision of technical procedures. The strongest relationship is when a GP is co-located with other GPs and other professionals (*p* < 0.001, Table 4 second column). When GPs are co-located with nurses, they are more likely to provide technical procedures compared to GPs co-located with other professionals (*p* < 0.001, see Appendix 3).

The use of guidelines is not associated with mono or multi-professional GP co-location (Table 4 third column), except when considering the multi-professional details as separate independent variables (see Appendix 3).

Compared with single-handed GP practices, mono and multi-professional GP co-location GP are associated with more collaboration with different providers (respectively *p* < 0.05 and *p* < 0.001), particularly when a GP is co-located with other GPs and other professionals (*p* < 0.001, Table 4 fourth column). More collaboration with other providers is also related to GP co-located with nurses, other health professionals and social workers, particularly GP co-location with a social worker (*p* < 0.001, see Appendix 3).

Compared with single handed GP practices, all co-located settings (only mono-professional, only multi-professional and mono and multi-professional) are more coordinated with secondary care (*p* < 0.001, Table 4, fifth column). GPs who are co-located with other professionals, in particular with specialists, midwives, physiotherapists, dentists or pharmacists, are more coordinated with secondary care (*p* < 0.01, see Appendix 3). These results are confirmed also after excluding GPs co-located with specialists (results not in tables) and therefore the positive association between GP co-location and integration with secondary care is not due only to the presence of specialists in the same practice/centre with GPs.

### Patient experience

We found that some areas of patient experience are strongly clustered at the country and GP levels. The intraclass correlation of both levels ranges from 60% for continuity of care to around 97% for accessibility and comprehensiveness (Table 5).

**Table 5:** Relationships between GP co-location and patient experience

Multilevel models (*p < 0.05; **p < 0.01; ***p < 0.001)	Accessibility ^a, b^		Continuity of care^a, b^		Comprehensiveness of care^a, b^	
*Fixed part*	*Coeff.*	*S.E.*	*Coeff.*	*S.E.*	*Coeff.*	*S.E.*
Single-handed GP practice (ref)						
- More GPs without other professionals	−0.359	0.366	−0.656	0.476	−0.825	0.587
- One GP with other professionals	−0.688	0.354	−1.352**	0.460	−1.439*	0.567
- More GPs with other professionals	−0.804*	0.340	−1.433**	0.443	−1.047	0.545
*Random part*	*Var.*	*S.E.*	*Var.*	*S.E.*	*Var.*	*S.E.*
Country level	32.312	8.061	80.276	19.940	86.434	21.970
GP level	44.180	0.797	64.054	1.351	114.423	2.050
Patient level	3.232	0.021	97.611	0.634	5.724	0.037
*Variance in empty model*	*Var.*	*S.E.*	*Var.*	*S.E.*	*Var.*	*S.E.*
Country level	35.020	8.586	100.427	24.559	96.802	23.721
GP level	46.587	0.804	72.022	1.438	114.199	1.965
Patient level	3.376	0.021	103.538	0.635	5.642	0.035
*Variance in model only with covariates*	*Var.*	*S.E.*	*Var.*	*S.E.*	*Var.*	*S.E.*
Country level	32.441	7.970	79.997	19.598	91.415	22.433
GP level	44.812	0.788	62.665	1.295	112.172	1.968
Patient level	3.286	0.021	95.480	0.607	5.605	0.036

^a^Covariates patient level: sex, age, education, household income, ethnicity, self-reported health status, main reason for visit, personal GP, chronic conditions

^b^Covariates GP level: sex, age, urbanisation, involvement in disease management, evening open time for GP practice, urbanisation

When GPs are co-located with other GPs and other professionals, patients experience less accessibility compared to patients of single-handed GP practices (*p* < 0.05, Table 5 second column). Also in the separate models, multi-professional GP co-location continues to be negatively associated with accessibility (p < 0.05, see Appendix 3). Co-location of a GP with social workers has a negative relationship with this patient experience (*p* < 0.01, see Appendix 3).

Compared to patients of GPs working in single-handed practices, patients perceive less continuity of care when their GPs are co-located only with other professionals or with other GPs and other professionals (*p* < 0.01, Table 5 third column). This negative association with continuity of care is also present as one of the main effects / as the main effect of GP co-location in a multi-professional setting (*p* < 0.01, see Appendix 3), and in practices where GPs are co-located with nurses, patients experience even less continuity of care (*p* < 0.001, see Appendix 3).

Compared with patients of GPs working in single-handed GP practices, patients perceive their GPs who are co-located only in multi-professional settings to provide less comprehensive care (*p* < 0.05, Table 5 fourth column). Comprehensiveness of care has the strongest negative relationship of GP co-location of all the dimensions of patient experiences analysed.

When we introduced GP outcome variables as covariates into the models analysing patient outcomes, the association between GP co-location and patient outcomes did not change significantly (results not in tables). This was particularly the case with variables such as provision of technical procedures, collaboration with different providers and coordination between GPs and secondary care which are positively related with GP co-location. The direction and strength of relationships which were statistically significant were confirmed. Only the negative associations with accessibility and comprehensiveness of care were slightly reduced when controlling for coordination with secondary care. When these models were stratified by the categorised GP outcome variables, the results were no longer interpretable.

The stratification by strength of primary care at a country level highlighted that in countries with a weak level of primary care, GP co-location is associated with worse patient experience, particularly when GPs are co-located in a multi-professional setting. In countries with a stronger level of primary care (medium and high level), the associations between GP co-location and patient experiences are not significant or, when significant, have a positive direction. In countries with a medium primary care structure, when GPs are co-located with other professionals and/or other GPs, patients perceive a better accessibility to GP care compared to a single-handed GP practice (Table 6).

**Table 6:** Interaction between the level of strength of primary care structure and GP co-location on patient experiences

Stratified multilevel models (*p < 0.05; **p < 0.01; ***p < 0.001)	Accessibility		Continuity of care		Comprehensiveness of care	
*Countries with weak PC structure*	*N*. = 14,973		*N*. = 14,920		*N*. = 14,917	
	*Coeff.*	*S.E.*	*Coeff.*	*S.E*.	*Coeff.*	*S.E.*
Single-handed GP practice (ref)						
- More GPs without other professionals	−0.766	0.918	−0.425	1.359	0.770	1.369
- One GP with other professionals	−2.637***	0.671	−2.681**	0.994	−2.269*	1.000
- More GPs with other professionals	−2.760***	0.709	−4.749***	1.051	−2.558*	1.057
Countries with medium PC structure	*N*. = 19,188		*N*. = 19.134		*N*. = 19,054	
	*Coeff.*	*S.E.*	*Coeff.*	*S.E.*	*Coeff.*	*S.E.*
Single-handed GP practice (ref)						
- More GPs without other professionals	0.927	0.482	−0.306	0.707	−1.175	0.850
- One GP with other professionals	0.822	0.526	−0.423	0.775	−0.139	0.928
- More GPs with other professionals	1.076*	0.517	0.610	0.763	−0.446	0.912
Countries with strong PC structure	*N*. = 19,830		*N*. = 19,733		*N*. = 19,648	
	*Coeff.*	*S.E.*	*Coeff.*	*S.E.*	*Coeff.*	*S.E.*
Single-handed GP practice (ref)						
- More GPs without other professionals	−0.922	0.649	0.147	0.534	−0.877	1.039
- One GP with other professionals	0.205	0.670	0.129	0.547	−1.835	1.072
- More GPs with other professionals	−0.566	0.593	−0.016	0.485	−0.101	0.919

## Discussion

This study reveals the relationships between GP co-location with other GPs and/or other professionals and outcomes at the GP level and patient experiences. The results highlight a positive association between GP co-location and GP outcomes, in terms of more technical procedures, and in terms of more collaboration with other primary and secondary care professionals. Conversely, GP co-location was negatively associated with some patient experiences, particularly in countries with a weak primary care, while in the other countries GP co-location was not associated or positively associated with patient outcomes.

The largest share of variability is at the GP level for almost all the GP outcomes, except for provision of technical procedures. The variations in patient perceptions of accessibility and comprehensiveness of care is due mainly to the GP and country levels, while continuity of care is more related to variability at the patient level. The country influence may play a different role in influencing the outcomes analysed from patient perspectives.

### Summary and interpretation of the relationships between GP co-location and GP outcomes

Almost all our hypotheses on GP outcomes concerning our first research question were confirmed. On the whole, from the point of view of GPs themselves, GP co-location has positive associations with a wider range of services provided, higher use of clinical governance tools and more inter-professional collaboration. The higher provision of technical procedures associated with GP co-location, particularly when they are with nurses, counteracts the notion that GPs have been gradually abandoning the technical aspects of medicine to specialists [59]. A recent study in the Netherlands found that patients prefer to substitute specialist care with GP care mainly for certain medical procedures (e.g. follow-up treatments and non-complex treatments) [58], and thus the broader provision of procedures by a GP co-located with other professionals can be seen as one aspect of a more responsive care approach. Indeed, more comprehensive primary care gives patients access to healthcare services at the primary care level for which they would usually have to go to other providers (in other locations).

This study highlights that GP co-location also has positive relationships with the collaboration with other professionals, confirming that physical proximity intensifies interactions and consequently informal and formal communication and knowledge exchange [17, 18]. GP co-location thus seems to be a first step to overcoming professional barriers and facilitating the consolidation of teamwork [15, 42, 49].

Co-located GPs tend to work more in coordination with secondary care which may be seen as positive in reducing fragmentation in healthcare pathways and ensuring a closer inter-professional cooperation [46]. The increased exchange of advice observed between GPs and specialists goes beyond the co-location of GPs with specialists. It therefore seems that co-location in itself involves organisational changes that then improve the connections between primary and secondary care, thus leading to a better coordination of patient care. However, this closer collaboration between GPs and specialists can be interpreted differently in different countries. Indeed, where specialists are employed by public health services, the increased collaboration with GPs is seen mainly in terms of a potential improvement in the appropriateness and integration of healthcare. On the other hand, where specialists are private professionals, the increased exchange of advice with GPs might be seen as a risk of encouraging more specialist care visits.

We found less strong evidence that co-located GPs use more clinical governance tools. The weak association of GP co-location with the use of guidelines does not seem to confirm the mutual influence of co-located professionals on the uptake of clinical governance tools, which was instead found by de Jong [13] in terms of mutual control in clinical practice. In any case, in completing the questionnaire, GPs may have been influenced by the differences in clinical guidelines in different countries [37].

### Summary and interpretation of the relationships between GP co-location and patient experience

Concerning the second research question, our hypotheses were almost completely refuted since we found that patients of co-located GPs did not perceive a better quality of care. Indeed, despite the general positive relationships between GP co-location and GP outcomes, GP co-location has a negative relationship with patient experiences in terms of accessibility, continuity and comprehensiveness of care.

These results can be interpreted in two ways. Although corresponding to improved healthcare service delivery from the providers’ perspective, GP co-location is not associated with improved patient experiences, because patients perceive better quality care when they are treated in smaller practices where they have an individual relationship with their own GP [10, 2]. An alternative interpretation is that the observed differences in GP outcomes due to co-location may not yet have led to effective teamwork, due to the challenges related to inter-professional collaboration, such as difficulties in overcoming barriers and conflicts or defining roles and common objectives [34, 41, 45]. In fact, another study underlined the importance of a multidisciplinary team combined with GP co-location in increasing patient satisfaction [6]. Both interpretations are in line with the discrepancy of perspectives between providers and users that Lloyd and Wait highlighted [38] in providing a definition of integrated care. The authors reported that users and providers may disagree as to whether or not a healthcare experience is really integrated. In this case and contrary to our hypothesis, GPs and patients do not experience GP co-location in the same way, maybe because the advantages of GP co-location for the former are not as important for the latter.

The results change when we take into account the differences among countries related to the strength of their primary care structure. Indeed, in countries with a medium or strong primary care, GP co-location is not associated with patient experiences or there are signs of positive associations with some patient experiences. At the same time in countries with a weaker primary care structure, GP co-location is related to very negative experiences from the patients’ perspective. Therefore, there is an interaction effect between GP co-location and the PC strength at the country level. This may be related to the different organisational models of GP co-location in different countries, from simple polyclinics to more integrated primary care centres, which may play a different role in affecting patient experience. In countries with a weak primary care, the organisational models of GP co-location may be implemented without a clear idea of the integration of services and professionals. Therefore patients perceived a lower quality of care in these settings compared to single-handed GP practices. Kringos et al. highlighted that teamwork and multidisciplinary collaboration have been poorly addressed in European primary care, especially in countries with weak primary care systems [33]. Not only do professionals need training to improve multidisciplinary collaboration, patients may also have difficulty in navigating larger facilities, where they deal with many different providers. In order to improve the patient experience, it may be necessary for larger facilities to be organised in a way that guarantees the continuity of care to patients.

### Summary and interpretation of the different relationships of mono- and multi-professional co-location of GPs and GP outcomes and patient experience

In order to answer the third research question, we focused on the differences in the relationships between the different components of GP co-location and GP outcomes and patient experiences. Mono-professional co-location has a less strong association with GP outcomes, while multi-professional co-location has a stronger association with GP outcomes but is negatively associated, or not associated at all, with patient experience.

Compared with single-handed GP practices, GPs co-located with other GPs and professionals, which constitute the most complex form of GP co-location, has the best impact on GP outcomes, while one GP with other professionals represents a very negative co-located setting in terms of patient experience, particularly regarding continuity and comprehensiveness of care.

Regarding GP outcomes, the provision of technical procedures is more strongly associated with the co-location of GPs with nurses. The collaboration with other providers has a stronger association with the co-location of GPs with social workers, while coordination with secondary care is mostly associated with the co-location of GPs with other health professionals, such as specialists, midwives, physiotherapists, dentists and pharmacists.

Regarding patient experiences, co-location of GPs with nurses has the strongest negative association with continuity of care, maybe because in this setting, the nurses are the first point of contact and play the role of primary caregiver in the relationship with patients. In fact, a Cochrane systematic review [35] reported that nurses tended to provide longer consultations than GPs and to take responsibility for the ongoing management of patients with particular chronic conditions. Therefore, when GPs are co-located with nurses, the GPs themselves may be less informed about the medical history or conditions in which patients live. Co-location of GPs with other health professionals has no relationship with patient experience, while co-location of GPs with social workers has a negative association with accessibility. These results can be explained by considering that firstly, the co-location of GPs with social workers is not very common and may also be more related to larger health centres with more limited accessibility.

### Strengths and limitations of the study

One major strength of our study is that it explores the relationships of GP co-location both with GP outcomes and patient experiences, providing evidence concerning the process and final outcomes of delivery changes in primary care through GP co-location. Other studies have included the analysis of the different perspectives related to GPs and patients, but with other approaches. Indeed, such studies have focused on GPs’ and patients’ perspectives regarding specific diseases [47] or concerning the quality of primary care [28, 43]. In these cases, the attention is on the juxtaposition between the clinical perspective and that of patients, focused more on the personal aspects of an illness, or between the professional and lay opinions in terms of what is valued in primary care.

In contrast, our study explored the GPs’ and patients’ perspectives in relation to different topics that are relevant for GPs and patients themselves. For GPs, we focused on the range of services provided, the use of clinical governance tools and inter-professional collaboration. For patients, we focused on the accessibility, comprehensiveness and continuity of care as perceived by the patients themselves. For both groups and their related topics of interest, we explored the correlation of these outcomes with a structural and organisational dimension of health services delivery (i.e. co-location of GPs in mono or multi-professionals settings).

The differences in the findings between the GPs’ and patients’ perspectives can be interpreted according to the Donabedian model [14], which focuses on the structure, process and outcomes of healthcare. According to this model, GP co-location can be considered as a structural factor of health services delivery, the topics which interest GPs are the process, whereas the topics of interest for patients are the final outcomes of the health services delivery. Therefore, the positive process (in terms of more technical procedures performed in GP practices, as well as more collaboration of GPs with other primary and secondary care professionals) due to the structural input of GP-colocation does not necessarily correspond to the final positive outcomes (in terms of more the accessibility, comprehensiveness and continuity of care perceived by patients). Indeed, the positive results of GP-colocation as perceived by GPs do not relate to positive results for patients, at least in the countries with a weak primary care, where the structural input of GP-colocation for health services delivery is probably characterised differently compared to countries with strong primary care.

An additional strength of the study is that the large number of countries involved enabled us to differentiate between the patient, practice and country levels and to look at the differences among countries based on their strength of primary care structure.

Our study also has limitations. GP co-location cannot be distinguished in terms of different organisational models and it has not been characterised according to the different composition of co-located teams. There is no information on the actual cooperation between co-located professionals, or on whether patients have actually experienced the inter-professional joint working in GPs co-location settings. Therefore, we cannot connect the structural and organisational dimension with professional integration. Moreover, considering that we used data from an international survey, GPs and patients from different countries may interpret the questions differently, also because of potential discrepancies related to different types of health systems among countries. As noted in another study on GP practice using the same data [22], some terms may have a different connotation from one country to another, in spite of the rigorous translation procedure implemented. Finally, GPs who are co-located with other GPs and/or other professionals may have consciously chosen this type of care setting, and therefore may be more biased towards co-location. This potential bias is perhaps less relevant for patients, as their freedom of choice for a specific practice may be influenced by the different regulatory and normative context existing in different countries, as well by the actual availability of different practices near the place they live.

## Conclusions

This article has shown that GP co-location is associated with a broader provision of health care services in a primary care setting and with more collaboration between GPs and other primary and secondary care professionals. In addition GP co-location is mostly related to negative patient experiences, however this relationship is conditioned by the strength of primary care structure at the country level. It is only in countries with weak primary care that patients perceived a worse quality of care in co-located services compared with single-handed GP practices.

Considering the high variability among countries of the diffusion of GP co-location, which probably also represents a different interest in and approach to this organisational strategy in primary care in different countries, further research should focus on specific models through which GP co-location with other professionals is organized. The organisational characteristics of GP co-location could thus be identified, as well as the kind of support from policies to co-located settings, which may consolidate the positive process of collaboration among different professionals and facilitate a positive patient experience.

## Appendix 1

**Table 7:** Data on health systems characteristics of the 34 countries involved in the study

Countries	Median number of extra professions in GP practice (apart from GPs) [22]	Model of healthcare system^a^	Role of GP as gatekeeper^a^	Overall Primary Care system strength [30–33]
Austria	1	Social health insurance (Bismarck)	No	Weak
Belgium	0	Social health insurance (Bismarck)	No	Strong
Bulgaria	1	Mixed model: tax-funded and social health insurance	Yes	Weak
Cyprus	6	Mixed model: tax-funded and private insurance	No, but planned	Weak
Czech Republic	1	Social health insurance (Bismarck)	Yes, partially	Medium
Denmark	2	Tax-funded (Beveridge)	Yes	Strong
Estonia	2	Social health insurance (Bismarck)	Yes, partially	Strong
Finland	7	Tax-funded (Beveridge)	Yes, partially	Strong
Germany	1	Social health insurance (Bismarck)	No	Medium
Greece	2	Tax-funded (Beveridge)	No	Weak
Hungary	1	Social health insurance (Bismarck)	Yes, partially	Weak
Iceland	5	Tax-funded (Beveridge)	Yes, partially	Weak
Ireland	3	Tax-funded (Beveridge)	Yes	Weak
Italy	1	Tax-funded (Beveridge)	Yes	Medium
Latvia	2	Tax-funded, but with high out-of-pocket payments	Yes	Medium
Lithuania	8	Transitional with National health insurance	Yes	Strong
Luxembourg	1	Social health insurance (Bismarck)	No	Weak
Malta	3	Tax-funded (Beveridge)	Yes	Weak
Netherlands	3	Social health insurance (Bismarck)	Yes	Strong
Norway	1	Tax-funded (Beveridge)	Yes	Medium
Poland	4	Transitional with National health insurance	Yes	Medium
Portugal	3	Tax-funded (Beveridge)	Yes	Strong
Romania	1	Transitional with Social health insurance	Yes	Medium
Slovakia	1	Social health insurance (Bismarck)	Yes, partially	Weak
Slovenia	4	Transitional with Social health insurance	Yes	Strong
Spain	6	Tax-funded (Beveridge)	Yes	Strong
Sweden	5	Tax-funded (Beveridge)	Yes, partially	Medium
Switzerland	1	Social health insurance (Bismarck)	No, but planned	Medium
Turkey	2	Social health insurance (Bismarck)	No	Weak
UK (England)	5	Tax-funded (Beveridge)	Yes	Strong
Australia	4	Tax-funded (Beveridge)	Yes	Strong
Canada	3	Tax-funded (Beveridge)	Yes	Strong
New Zealand	4	Tax-funded (Beveridge)	Yes	Strong
FYR Macedonia	1	Social health insurance (Bismarck)	Yes	Medium

^a^The information were extracted from the last published countries reports “Health Systems in Transition” of European Observatory on Health Systems and Policies

The table shows for each country involved in the study the median number of extra professions in GP practice (apart from GPs), the model of healthcare system, the role of GP as gatekeeper, the overall primary care system strength.

## Appendix 2

**Table 8:** Distribution of dependent variables at the GP level by country

Countries	Provision of technical procedures^a^ *N* = 7136 Mean (SD)	Use of guidelines^b^ *N* = 7127 Mean (SD)	Collaboration with different providers^c^ *N* = 7145 Mean (SD)	Coordination with secondary care^d^ *N* = 7108 Mean (SD)
Austria	2.062 (0.568)	0.843 (0.271)	1.716 (0.256)	1.721 (0.419)
Belgium	2.373 (0.499)	0.899 (0.197)	1.875 (0.279)	1.704 (0.522)
Bulgaria	1.762 (0.410)	0.747 (0.323)	2.078 (0.302)	1.940 (0.554)
Cyprus	1.260 (0.199)	0.516 (0.393)	2.050 (0.289)	1.541 (0.490)
Czech Republic	1.396 (0.291)	0.838 (0.249)	1.832 (0.229)	1.657 (0.396)
Denmark	2.538 (0.452)	0.923 (0.143)	1.560 (0.184)	1.638 (0.357)
Estonia	1.551 (0.437)	0.309 (0.183)	1.964 (0.218)	1.605 (0.386)
Finland	3.312 (0.481)	0.958 (0.114)	2.103 (0.195)	1.918 (0.394)
Germany	1.832 (0.464)	0.920 (0.186)	1.576 (0.232)	1.797 (0.409)
Greece	2.428 (0.457)	0.905 (0.187)	2.090 (0.244)	1.734 (0.406)
Hungary	1.366 (0.328)	0.698 (0.347)	2.037 (0.235)	1.452 (0.344)
Iceland	2.887 (0.502)	0.780 (0.295)	2.064 (0.197)	1.927 (0.465)
Ireland	2.680 (0.551)	0.875 (0.203)	1.990 (0.225)	1.492 (0.350)
Italy	1.392 (0.306)	0.869 (0.228)	1.698 (0.246)	1.570 (0.431)
Latvia	1.426 (0.303)	0.921 (0.182)	2.075 (0.226)	1.869 (0.424)
Lithuania	1.277 (0.297)	0.879 (0.152)	2.056 (0.252)	1.669 (0.453)
Luxembourg	2.081 (0.471)	0.874 (0.263)	1.681 (0.306)	1.723 (0.438)
Malta	2.203 (0.469)	0.864 (0.244)	1.907 (0.252)	1.551 (0.450)
Netherlands	3.287 (0.391)	0.959 (0.112)	2.121 (0.220)	2.062 (0.452)
Norway	3.123 (0.442)	0.898 (0.188)	1.853 (0.222)	2.011 (0.419)
Poland	1.325 (0.313)	0.891 (0.237)	2.097 (0.267)	1.495 (0.471)
Portugal	1.782 (0.422)	0.908 (0.178)	1.978 (0.207)	1.359 (0.309)
Romania	1.428 (0.360)	0.615 (0.367)	1.882 (0.274)	1.582 (0.483)
Slovakia	1.300 (0.346)	0.488 (0.332)	1.806 (0.243)	1.625 (0.483)
Slovenia	1.669 (0.465)	0.959 (0.102)	2.024 (0.196)	1.558 (0.353)
Spain	2.212 (0.488)	0.938 (0.156)	1.735 (0.240)	1.479 (0.427)
Sweden	2.990 (0.367)	0.919 (0.198)	2.123 (0.242)	1.663 (0.370)
Switzerland	2.580 (0.587)	0.794 (0.315)	1.716 (0.241)	1.882 (0.370)
Turkey	1.749 (0.452)	0.654 (0.376)	1.802 (0.253)	1.463 (0.406)
UK (England)	2.642 (0.505)	0.989 (0.016)	2.077 (0.198)	1.683 (0.409)
Australia	2.887 (0.590)	0.805 (0.309)	1.824 (0.256)	1.614 (0.386)
Canada	2.648 (0.606)	0.922 (0.161)	1.971 (0.296)	1.942 (0.492)
New Zealand	3.220 (0.488)	0.792 (0.303)	1.900 (0.211)	1.690 (0.380)
FYR Macedonia	1.277 (0.216)	0.924 (0.205)	1.900 (0.246)	1.726 (0.470)
**Average**	**2.134 (0.794)**	**0.854 (0.260)**	**1.909 (0.291)**	**1.695 (0.467)**
Weak PC countries	1.795 (0.671)	0.766 (0.320)	1.950 (0.294)	1.684 (0.471)
Medium PC countries	2.156 (0.818)	0.876 (0.234)	1.907 (0.288)	1.725 (0.458)
Strong PC countries	2.337 (0.771)	0.890 (0.222)	1.885 (0.289)	1.675 (0.472)

^a^Scale 1 to 4

^b^Scale 0 to 1

^c^Scale 1 to 3

^d^Scale 1 to 3

The table shows for each country the mean and standard deviation of the dependent variables at the GP level, such as the provision of technical procedures, use of guidelines, collaboration with different providers, coordination with secondary care

## Appendix 3

**Table 9:** Detailed results of the multilevel models of GP co-location (mono-professional – model 1, multi-professional - model 2, interaction between mono and multi-professional – model 3, details of multi-professional – model 4) and GP outcomes

Multilevel models (**p* < 0.05; ***p* < 0.01; ****p* < 0.001)		Provision of technical procedures^a^		Use of guidelines^a^		Collaboration with different providers^a^		Integration with secondary care^a^	
		*N*. = 7136		*N*. = 7127		*N*. = 7145		*N*. = 7108	
Empty model	*Fixed part*	*Coeff.*	*S.E.*	*Coeff.*	*S.E.*	*Coeff.*	*S.E.*	*Coeff.*	*S.E.*
	Intercept	2.116***	0.115	0.843***	0.021	1.916***	0.027	1.687***	0.030
	*Random part*	*Var.*	*S.E.*	*Var.*	*S.E.*	*Var.*	*S.E.*	*Var.*	*S.E.*
	Country level	0.452	0.110	0.014	0.004	0.025	0.006	0.029	0.007
	GP level	0.201	0.003	0.054	0.001	0.060	0.001	0.186	0.003
	ICC								
	Country	69.2%		20.6%		29.6%		13.4%	
	GP	30.8%		79.4%		70.4%		86.6%	
		*N*. = 6934		*N*. = 6940		*N*. = 6944		*N*. = 6908	
Model only covariates	Fixed part	Coeff.	S.E.	Coeff.	S.E.	Coeff.	S.E.	Coeff.	S.E.
	Intercept	2.034***	0.110	0.790***	0.020	1.873***	0.029	1.593***	0.033
	Random part	Var.	S.E.	Var.	S.E.	Var.	S.E.	Var.	S.E.
	Country level	0.409	0.099	0.012	0.003	0.027	0.007	0.029	0.007
	GP level	0.185	0.003	0.053	0.001	0.058	0.001	0.182	0.003
	*ICC*								
	Country	68.8%		18.8%		31.9%		13.9%	
	GP	31.2%		81.2%		68.1%		86.1%	
		*N*. = 6655		*N*. = 6660		*N*. = 6663		*N*. = 6628	
MODEL 1 (variable 1)	*Fixed part*	*Coeff.*	*S.E.*	*Coeff.*	*S.E.*	*Coeff.*	*S.E.*	*Coeff.*	*S.E.*
	Intercept	1.985***	0.111	0.782***	0.021	1.844***	0.031	1.600***	0.033
	GP co-located with other GP	0.093***	0.013	0.007	0.007	0.047***	0.007	0.011	0.013
	Random part	Var.	S.E.	Var.	S.E.	Var.	S.E.	Var.	S.E.
	Country level	0.396	0.098	0.012	0.003	0.028	0.007	0.028	0.007
	GP level	0.184	0.003	0.053	0.001	0.058	0.001	0.185	0.003
	*ICC*								
	Country	68.3%		18.7%		32.6%		13.0%	
	GP	31.7%		81.3%		67.4%		87.0%	
		*N*. = 6925		*N*. = 6931		*N*. = 6935		*N*. = 6899	
MODEL 2 (variable 2)	*Fixed part*	*Coeff.*	*S.E.*	*Coeff.*	*S.E.*	*Coeff.*	*S.E.*	*Coeff.*	*S.E.*
	Intercept	1.944***	0.112	0.799***	0.022	1.782***	0.028	1.553***	0.036
	GP co-located with other professional	0.113***	0.018	−0.011	0.009	0.117***	0.010	0.052**	0.018
	Random part	Var.	S.E.	Var.	S.E.	Var.	S.E.	Var.	S.E.
	Country level	0.411	0.100	0.012	0.003	0.022	0.005	0.031	0.008
	GP level	0.184	0.003	0.053	0.001	0.057	0.001	0.182	0.003
	*ICC*								
	Country	69.1%		18.6%		27.6%		14.5%	
	GP	30.9%		81.4%		72.4%		85.5%	
		*N*. = 6655		*N*. = 6660		*N*. = 6663		*N*. = 6628	
MODEL 3 (interaction)	*Fixed part*	*Coeff.*	*S.E.*	*Coeff.*	*S.E.*	*Coeff.*	*S.E.*	*Coeff.*	*S.E.*
	Intercept	1.885***	0.113	0.788***	0.023	1.768***	0.029	1.528***	0.037
	Single-handed GP practice (ref)								
	- More GPs without other professionals	0.153***	0.023	0.021	0.013	0.033*	0.013	0.090***	0.024
	- One GP with other professionals	0.146***	0.022	−0.003	0.012	0.106***	0.013	0.108***	0.022
	- More GPs with other professionals	0.200***	0.021	−0.001	0.012	0.143***	0.012	0.080***	0.022
	*Random part*	*Var.*	*S.E.*	*Var.*	*S.E.*	*Var.*	*S.E.*	*Var.*	*S.E.*
	Country level	0.415	0.100	0.012	0.003	0.023	0.006	0.028	0.007
	GP level	0.182	0.003	0.053	0.001	0.057	0.001	0.184	0.003
	ICC								
	Country	69.0%		18.5%		28.5%		13.4%	
	GP	31.0%		81.5%		71.5%		86.6%	
		*N*. = 6813		*N*. = 6819		*N*. = 6823		*N*. = 6787	
MODEL 4 (var 2 details)	Fixed part	Coeff.	S.E.	Coeff.	S.E.	Coeff.	S.E.	Coeff.	S.E.
	Intercept	1.931***	0.111	0.787***	0.022	1.756***	0.026	1.544***	0.036
	GP with nurse	0.103***	0.018	−0.004	0.010	0.093***	0.010	0.037*	0.018
	GP with other health professional	0.060**	0.014	0.003*	0.007	0.079***	0.008	0.043**	0.014
	GP with social worker	0.043**	0.018	0.007	0.010	0.118***	0.010	0.022	0.018
	*Random part*	*Var.*	*S.E.*	*Var.*	*S.E.*	*Var.*	*S.E.*	*Var.*	*S.E.*
	Country level	0.406	0.099	0.012	0.003	0.019	0.005	0.031	0.008
	GP level	0.182	0.003	0.053	0.001	0.054	0.001	0.181	0.003
	ICC								
	Country	69.0%		18.7%		26.3%		14.7%	
	GP	31.0%		81.3%		73.6%		85.3%	

^a^Covariates GP level: sex, age, urbanisation, involvement in disease management

**Table 10:** Detailed results of the multilevel models of GP co-location (mono-professional – model 1, multi-professional - model 2, interaction between mono and multi-professional – model 3, details of multi-professional – model 4) and patient experience

Multilevel models (**p* < 0.05; ***p* < 0.01; ****p* < 0.001)		Accessibility ^a, b^		Continuity of care^a, b^		Comprehensiveness of care ^a, b^	
		*N*. = 60,309		*N*. = 60,069		*N*. = 59,851	
Empty model	*Fixed part*	*Coeff.*	*S.E.*	*Coeff.*	*S.E.*	*Coeff.*	*S.E.*
	Intercept	85.141***	1.019	90.439***	1.723	68.934***	1.694
	*Random part*	*Var.*	*S.E.*	*Var.*	*S.E.*	*Var.*	*S.E.*
	Country level	35.020	8.586	100.427	24.559	96.802	23.721
	GP level	46.587	0.804	72.022	1.438	114.199	1.965
	Patient level	3.376	0.021	103.538	0.635	5.642	0.035
	*ICC*						
	Country	41.2%		36.4%		44.7%	
	GP	54.8%		26.1%		52.7%	
		*N*. = 56,217		*N*. = 56,049		*N*. = 55,879	
Model only covariates	*Fixed part*	*Coeff.*	*S.E.*	*Coeff.*	*S.E.*	*Coeff.*	*S.E.*
	Intercept	82.920***	1.011	77.096***	1.599	65.754***	1.690
	*Random part*	*Var.*	*S.E.*	*Var.*	*S.E.*	*Var.*	*S.E.*
	Country level	32.441	7.970	79.997	19.598	91.415	22.433
	GP level	44.812	0.788	62.665	1.295	112.172	1.968
	Patient level	3.286	0.021	95.480	0.607	5.605	0.036
	*ICC*						
	Country	40.3%		33.6%		43.7%	
	GP	55.6%		26.3%		53.6%	
		*N*. = 53,951		*N*. = 53,787		*N*. = 53,619	
MODEL 1 (variable 1)	*Fixed part*	*Coeff.*	*S.E.*	*Coeff.*	*S.E.*	*Coeff.*	*S.E.*
	Intercept	83.261***	1.037	77.016***	1.636	65.399***	1.698
	GP co-located with other GP	−0.251	0.212	−0.385	0.276	−0.070	0.341
	Random part	Var.	S.E.	Var.	S.E.	Var.	S.E.
	Country level	32.718	8.158	80.441	20.008	87.367	21.790
	GP level	44.408	0.798	64.169	1.353	114.536	2.052
	Patient level	3.232	0.021	97.611	0.634	5.724	0.037
	ICC						
	Country	40.7%		33.2%		42.1%	
	GP	55.3%		26.5%		55.2%	
		*N*. = 56,165		*N*. = 55,998		*N*. = 55,827	
MODEL 2 (variable 2)	Fixed part	Coeff.	S.E.	Coeff.	S.E.	Coeff.	S.E.
	Intercept	83.379***	1.027	77. 974***	1.620	66.293***	1.746
	GP co-located with other professional	−0.593	0.278	−1.101**	0.357	−0.713	0.440
	*Random part*	*Var.*	*S.E.*	*Var.*	*S.E.*	*Var.*	*S.E.*
	Country level	32.004	7.867	79.720	19.531	90.662	22.556
	GP level	44.898	0.787	62.542	1.293	112.126	1.968
	Patient level	3.286	0.021	95.379	0.607	5.607	0.036
	*ICC*						
	Country	40.0%		33.5%		43.5%	
	GP	55.9%		26.3%		53.8%	
		*N*. = 53,951		*N*. = 53,787		*N*. = 53,619	
MODEL 3 (interaction)	*Fixed part*	*Coeff.*	*S.E.*	*Coeff.*	*S.E.*	*Coeff.*	*S.E.*
	Intercept	83.727***	1.057	77.935***	1.663	66.345***	1.724
	Single-handed GP practice (ref)						
	- More GPs without other professionals	−0.359	0.366	−0.656	0.476	−0.825	0.587
	- One GP with other professionals	− 0.688	0.354	−1.352**	0.460	−1.439*	0.567
	- More GPs with other professionals	−0.804*	0.340	−1.433**	0.443	−1.047	0.545
	*Random part*	*Var.*	*S.E.*	*Var.*	*S.E.*	*Var.*	*S.E.*
	Country level	32.312	8.061	80.276	19.940	86.434	21.970
	GP level	44.180	0.797	64.054	1.351	114.423	2.050
	Patient level	3.232	0.021	97.611	0.634	5.724	0.037
	*ICC*						
	Country	40.4%		33.2%		41.8%	
	GP	55.5%		26.5%		55.4%	
		*N*. = 55,251		*N*. = 55,086		*N*. = 54,916	
MODEL 4 (var 2 details)	*Fixed part*	*Coeff.*	*S.E.*	*Coeff.*	*S.E.*	*Coeff.*	*S.E.*
	Intercept	83.452***	1.027	78.161***	1.680	66.322***	1.731
	GP with nurse	−0.563	0.289	−1.386***	0.370	−0.860	0.456
	GP with other health professional	−0.025	0.227	0.286	0.290	−0.067	0.358
	GP with social worker	−0.818**	0.304	−0.273	0.388	0.475	0.480
	Random part	Var.	S.E.	Var.	S.E.	Var.	S.E.
	Country level	31.915	7.825	86.316	21.155	92.273	22.657
	GP level	44.895	0.797	61.916	1.292	112.850	1.980
	Patient level	3.290	0.021	95.114	0.610	5.611	0.036
	ICC						
	Country	39.8%		35.5%		44.0%	
	GP	56.0%		25.4%		53.3%	

^a^Covariates patient level: sex, age, education, household income, ethnicity, self-reported health status, main reason for visit, personal GP, chronic conditions

^b^Covariates GP level: sex, age, urbanisation, involvement in disease management, evening open time for GP practice, urbanisation

The tables show the results of the multilevel linear regression analysis performed in the study, with the details for each model:

- empty model
- model with only covariates
- model 1 exploring the main effects of the mono-professional GP co-location
- model 2 exploring the main effects of the multi-professional GP co-location
- model 3 exploring the interaction of mono e multi-professional GP co-location
- model 4 exploring the main effect of the three sub-variables of multi-professional GP co-location, such as co-location with nurses, with other health professionals and with social workers

In particular the first table shows the results of the multilevel models analysing the relationships of GP co-location and GP outcomes and the second table shows the results of the multilevel models analysing the relationships of GP co-location and patient experiences.

## Abbreviations

GP: General practitioner
QUALICOPC: Quality and Costs of Primary Care in Europe
